# Supplementary material for: Application of a Latent Transition Model to Estimate the Usual Prevalence of Dietary Patterns
Source: Nutrients. 2020 Dec 31;13(1):133. doi: 10.3390/nu13010133 (PMC7823363; doi:10.3390/nu13010133)
Supplement: Supplementary file 1 [file nutrients-13-00133-s001.pdf]

Supplementary Material

Figure S1: Flowchart of participant's selection.

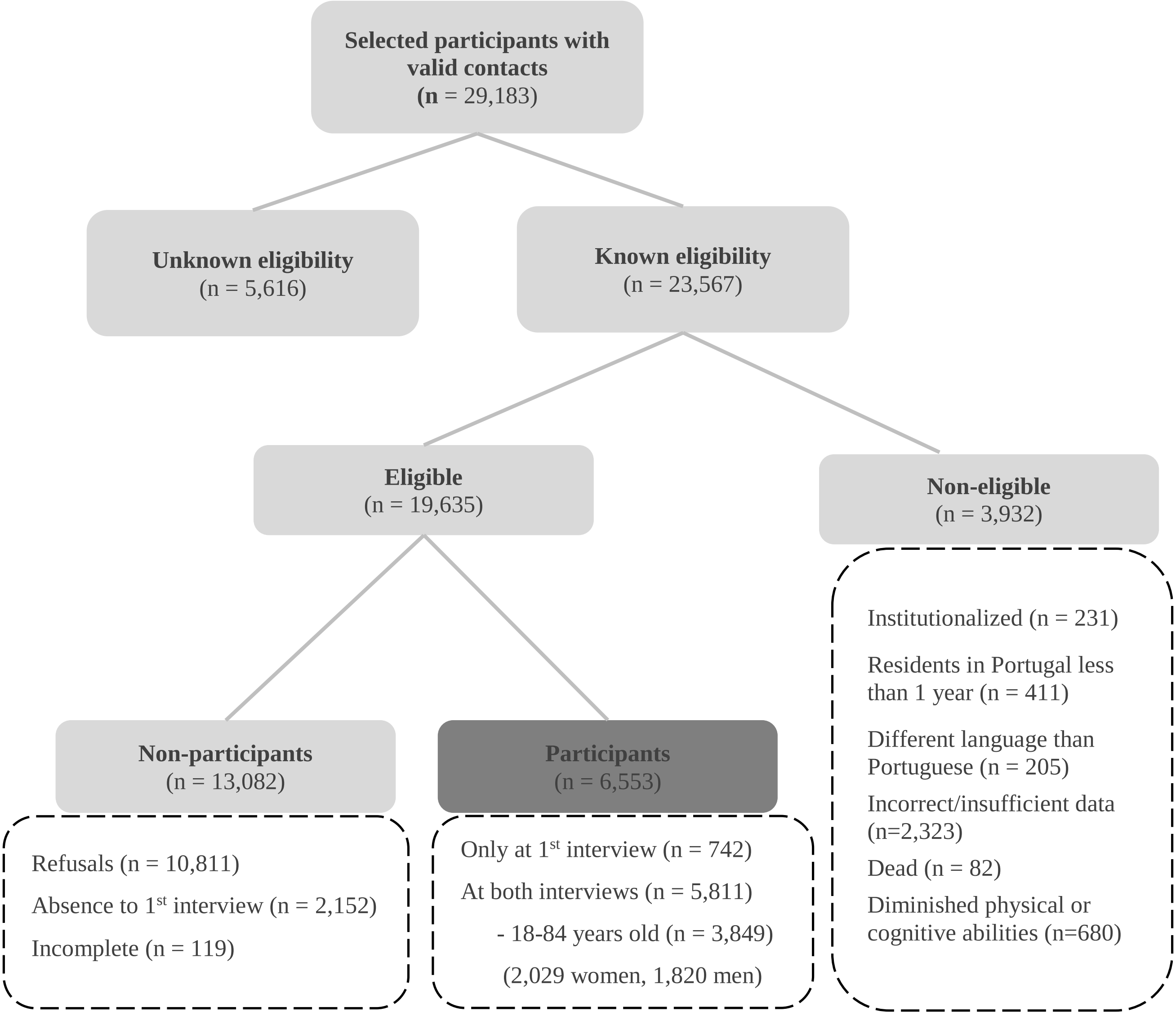

Table S1 Food groups' description

**Food groups**	**Food items**	**Minimum intake[a] (g/day)**	**Median intake[a] (g/day)**	**Maximum intake[a] (g/day)**	**ICC (95%CI) [b]**
vegetables		0.42	86.6	1237.0	0.49 (0.46; 0.52)
fruits		1.55	155.4	1489.6	0.66 (0.64; 0,67)
legumes		2.88	55.2	652.6	0.11 (0.06; 0.16)
vegetable soup		7.89	141.6	1132.6	0.52 (0.49; 0.64)

dairy	milk, yogurts, cheese, milk shakes	3.98	246.5	2111.0	0.74 (0.72; 0.75)
cereals and tubers	pasta, rice, potatoes and other tubers	4.26	124.4	1362.2	0.41 (0.37; 0.44)
bread		4.0	97.0	1303.0	0.61 (0.59; 0.63)
breakfast cereals		1.6	45.0	600.0	0.58 (0.56; 0.61)
white meat		2.33	141.4	1414.3	0.25 (0.21; 0.29)
red meat		3.52	120.0	1663.7	0.23 (0.19; 0.27)
processed meat		2.20	26.0	517.7	0.27 (0.23; 0.30)
fishery	fish and seafood	1.03	83.8	1043.3	0.18 (0.14; 0.22)
eggs		3.14	59.0	576.2	0.20 (0.16; 0.24)
salty snacks	salty snacks, pizzas, chips	0.00	110.0	1576.8	0.13 (0.08; 0.17)
olive oil		0.92	13.5	55.1	0.30 (0.27; 0.34)
butter		1.25	10.0	158.1	0.53 (0.51; 0.56)
margarines		1.87	10.0	90.0	0.63 (0.61; 0.65)
sweets	sweets, cakes, cookies	0.90	43.4	1592.4	0.34 (0.31; 0.38)
table sugar		0.90	9.0	405.0	0.60 (0.57; 0.62)
artificial sweeteners		0.50	2.0	47.4	0.78 (0.77; 0.79)
wine		1.68	200.0	1980.0	0.79 (0.78; 0.80)
beer		15.1	333.3	5302.5	0.47 (0.44; 0.50)
soft drinks		6.0	326.0	2221.8	0.67 (0.65; 0.68)
nectars		4.0	212.0	1575.0	0.43 (0.40; 0.46)
natural fruit juices		1.1	172.2	1953.0	0.33 (0.29; 0.36)
coffee		0.20	56.1	1656.5	0.72 (0.71; 0.74)
teas		1.0	312.0	3400.0	0.70 (0.68; 0.71)
water		1.0	998.0	7984.0	0.74 (0.73; 0.76)

ICC: Intraclass correlation coefficient; 95%CI: 95% confidence intervals

[a] Intake among the consumers (not considering those with a nil intake).
[b] Intra-class correlation coefficient (ICC) and the respective 95% confidence intervals, measuring the inter-person variability of dietary intake of each food group.

Table S2 Probabilities of food group consumption[a] conditional on a dietary pattern derived by the latent class model with concomitant variables (age and sex)[1]

		nil	≤median	>median		nil	≤median	>median
DP1	vegetables	16.6%	48.2%	35.3%	fishery	47.9%	31.1%	21.0%
DP2		9.4%	35.6%	**55.0%**		25.5%	26.5%	**48.1%**
DP3		13.4%	66.7%	19.8%		45.7%	37.8%	16.5%
DP4		10.9%	42.6%	46.5%		25.3%	30.6%	44.0%

DP5		9.6%	47.9%	42.5%		41.2%	30.3%	28.6%
DP6		0.0%	20.5%	**79.5%**		16.3%	37.0%	**46.6%**
DP1	fruits	23.9%	51.9%	24.2%	salty snacks	55.3%	19.2%	**25.5%**
DP2		7.4%	31.6%	**61.0%**		87.8%	6.5%	5.7%
DP3		9.3%	49.4%	41.4%		84.0%	9.3%	6.7%
DP4		12.6%	35.4%	**52.1%**		86.6%	7.1%	6.2%
DP5		20.2%	53.7%	26.2%		60.8%	19.5%	**19.7%**
DP6		1.9%	32.9%	**65.2%**		86.3%	6.7%	7.1%
DP1	legumes	84.2%	7.5%	8.3%	olive oil	85.6%	10.0%	4.4%
DP2		74.7%	10.7%	**14.6%**		65.7%	16.6%	17.6%
DP3		78.3%	16.7%	5.0%		73.7%	18.5%	7.8%
DP4		68.7%	13.6%	**17.8%**		62.6%	21.5%	16.0%
DP5		74.4%	12.0%	13.6%		78.1%	15.0%	6.9%
DP6		76.5%	10.4%	13.1%		41.6%	32.4%	**26.1%**
DP1	vegetable soup	56.3%	23.0%	20.8%	sweets	12.7%	27.7%	**59.6%**
DP2		33.7%	24.7%	**41.6%**		**34.4%**	36.0%	29.6%
DP3		18.6%	36.8%	**44.6%**		4.5%	57.5%	38.0%
DP4		38.2%	27.4%	**34.5%**		0.0%	62.9%	37.1%
DP5		55.3%	28.1%	16.6%		0.0%	33.7%	**66.3%**
DP6		38.2%	35.4%	26.5%		13.4%	51.5%	35.1%
DP1	dairy	8.2%	43.4%	48.4%	table sugar	**83.5%**	13.4%	3.1%
DP2		11.5%	45.8%	42.7%		**100.0%**	0.0%	0.0%
DP3		8.3%	42.5%	49.3%		35.5%	34.0%	30.5%
DP4		16.3%	47.4%	36.2%		0.0%	49.0%	**51.0%**
DP5		4.2%	47.4%	48.3%		15.2%	33.7%	**51.1%**
DP6		8.4%	46.1%	45.5%		63.8%	26.2%	10.0%
DP1	cereals and tubers	4.8%	42.1%	53.1%	artificial sweeteners	99.9%	0.0%	0.2%
DP2		11.1%	37.4%	51.5%		73.6%	11.2%	**15.2%**
DP3		11.1%	63.4%	25.6%		91.4%	5.5%	3.1%
DP4		4.8%	33.7%	**61.6%**		94.8%	3.1%	2.1%
DP5		2.1%	39.1%	**58.9%**		96.1%	2.1%	1.8%
DP6		11.7%	54.8%	33.5%		88.8%	7.1%	4.1%
DP1	bread	11.1%	63.0%	25.9%	wine	84.1%	12.6%	3.3%
DP2		2.3%	29.8%	**68.0%**		31.6%	21.7%	**46.7%**
DP3		2.2%	57.8%	39.9%		76.7%	19.8%	3.5%
DP4		2.3%	32.1%	**65.6%**		16.3%	18.4%	**65.3%**
DP5		2.8%	46.3%	51.0%		63.9%	23.9%	12.2%
DP6		9.2%	54.7%	36.2%		73.1%	21.5%	5.5%
DP1	breakfast cereals	53.1%	16.0%	**30.9%**	beer	90.9%	4.9%	4.2%
DP2		84.9%	6.6%	8.6%		77.1%	12.0%	10.9%
DP3		80.6%	12.3%	7.0%		98.8%	1.2%	0.0%
DP4		92.0%	3.3%	4.7%		69.7%	15.3%	15.0%
DP5		74.2%	13.4%	12.5%		76.4%	11.0%	12.6%
DP6		65.3%	20.3%	14.4%		97.4%	2.2%	0.3%
DP1	white meat	34.4%	25.0%	**40.6%**	soft drinks	44.5%	18.1%	**37.4%**
DP2		53.1%	21.9%	25.1%		80.5%	9.9%	9.6%
DP3		49.6%	35.4%	15.0%		79.3%	15.5%	5.2%
DP4		48.8%	22.4%	28.9%		80.9%	12.8%	6.4%
DP5		40.2%	26.9%	32.9%		34.8%	26.8%	**38.3%**
DP6		48.2%	26.1%	25.7%		86.8%	10.8%	2.4%
DP1	red meat	29.7%	30.5%	39.8%	coffee	71.7%	23.6%	4.8%
DP2		35.8%	27.8%	36.4%		25.2%	35.4%	39.4%
DP3		39.5%	44.7%	15.8%		10.4%	49.6%	40.0%
DP4		26.8%	31.4%	41.8%		2.3%	51.5%	46.2%

DP5		19.0%	31.0%	**49.9%**		0.8%	42.4%	**56.8%**
DP6		49.5%	30.5%	20.0%		20.1%	37.6%	42.3%
DP1	processed meat	47.8%	23.4%	28.9%	teas	85.0%	9.1%	5.9%
DP2		52.9%	19.8%	27.3%		77.9%	12.0%	10.1%
DP3		69.6%	21.0%	9.4%		47.1%	26.0%	**26.9%**
DP4		52.9%	19.7%	27.5%		79.8%	13.0%	7.2%
DP5		32.2%	30.4%	**37.4%**		83.4%	10.2%	6.5%
DP6		64.8%	23.3%	11.9%		54.5%	16.6%	**28.9%**

[1] Food group consumption divided into three categories: nil intake, equal/below and above median intake. Some food groups were not presented as their distributions were very similar across dietary patterns: eggs, butter, margarines, nectars, natural fruit juices and water.

[b] DP1 – 9.7%; DP2 – 9.7%; DP3 – 21.3%; DP4 – 18.5%; DP5 – 24.8%; DP6 – 16.0% (BIC: 179039). Major differences are in bold-type.
